# Engineering peptide-modified alginate-based bioinks with cell-adhesive properties for biofabrication†

**DOI:** 10.1039/d3ra08394b

**Published:** 2024-04-26

**Authors:** Emine Karakaya, Luisa Gleichauf, Lisa Schöbel, Ahmed Hassan, Anahita Ahmadi Soufivand, Joerg Tessmar, Silvia Budday, Aldo R. Boccaccini, Rainer Detsch

**Affiliations:** a Department of Material Science and Engineering, Institute for Biomaterials, Friedrich-Alexander University Erlangen-Nuremberg Germany rainer.detsch@fau.de; b Department of Mechanical Engineering, Institute of Continuum Mechanics and Biomechanics, Friedrich-Alexander-University Erlangen-Nuremberg Germany; c Department for Functional Materials in Medicine and Dentistry, University of Würzburg Germany

## Abstract

Alginate (ALG) and its oxidised form alginate-dialdehyde (ADA) are highly attractive materials for hydrogels used in 3D bioprinting as well as drop-on-demand (DoD) approaches. However, both polymers need to be modified using cell-adhesive peptide sequences, to obtain bioinks exhibiting promising cell-material interactions. Our study explores the modification of ALG- and ADA-based bioinks with the adhesive peptides YIGSR (derived from laminin), RRETEWA (derived from fibronectin) and IKVAV (derived from laminin) for 3D bioprinting. Two coupling methods, carbodiimide and Schiff base reactions, were employed to modify the polymers with peptides. Analytical techniques, including FTIR and NMR were used to assess the chemical composition, revealing challenges in confirming the presence of peptides. The modified bioinks exhibited decreased stability, viscosity, and stiffness, particularly-ADA-based bioinks in contrast to ALG. Sterile hydrogel capsules or droplets were produced using a manual manufacturing process and DoD printing. NIH/3T3 cell spreading analysis showed enhanced cell spreading in carbodiimide-modified ADA, Schiff base-modified ADA, and PEG-Mal-modified ADA. The carbodiimide coupling of peptides worked for ADA, however the same was not observed for ALG. Finally, a novel mixture of all used peptides was evaluated regarding synergistic effects on cell spreading which was found to be effective, showing higher aspect ratios compared to the single peptide coupled hydrogels in all cases. The study suggests potential applications of these modified bioinks in 3D bioprinting approaches and highlights the importance of peptide selection as well as their combination for improved cell–material interactions.

## Abstract 2

Alginate-based bioinks used for DoD approaches can be modified with specific peptide-containing components so that incorporated printed cells show significantly higher cell elongation ratios after 7 days (created with https://www.BioRender.com).

## Graphical abstract

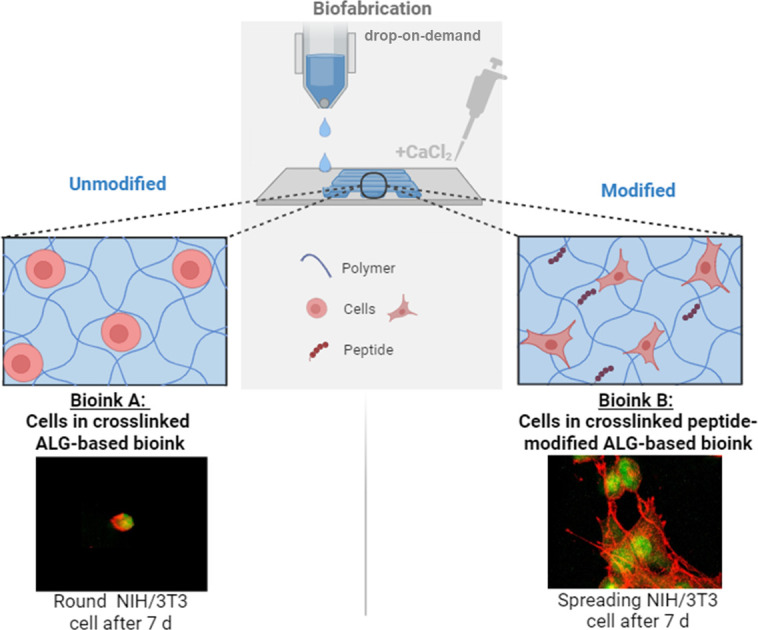

## 1. Introduction

Hydrogels are stable networks of synthetic or natural polymers, which can be crosslinked in different ways, *via* chemical (*i.e.* Schiff base) or physical linkages (*i.e.* ionically *via* divalent cations), to form stable three-dimensional (3D) constructs.^1–3^ They are particularly interesting for biofabrication since these materials can mimic the natural extracellular matrix (ECM) due to their high water content and promising physical properties.^4^ In this regard, the hydrogel should fulfil both requirements: (i) mimicking the natural ECM, which includes many biophysical and biochemical signals, and (ii) providing biomechanical support for cells. It is well known that alginate (ALG) hydrogels provide a certain protection and mechanical support for cells. However, no specific biochemical signal is transmitted,^5–7^ because ALG does not interact with cells to promote their attachment to the material. To introduce this interaction, ALG must be modified with cell-adhesive sequences to achieve interactions with cells.^8^ To enable these polymer modifications, native ALG is commonly oxidised to alginate-dialdeyhde (ADA) and later on combined with natural cell-attractive polymers, *i.e.* gelatine (GEL), promoting the desired cell–material interactions.^9,10^ Another approach is to introduce synthetic small peptide sequences directly into the polymer chain to overcome the challenges of natural compounds like GEL, for example, batch to batch differences or thermo-responsive behaviour.^11^ As stated in the literature, the synthesis of peptide-modified ALG and ADA is commonly achieved by a carbodiimide reaction.^12–14^ However, this pathway shows some drawbacks due to the time-consuming purification, toxic reactants, side products, and its questionable efficiency.^15^ Another approach is to introduce peptides into ADA by the reversible binding of peptide-containing compounds *via* Schiff-base formation.^8,16^ This method does not require any additional reactants or further purification steps and can therefore be considered as a one pot preparation method.^17^ However, the mentioned procedure has rarely been done before and it has never been compared to the carbodiimide reaction in terms of efficiency and substitution degree. Moreover, not only methods for the introduction of peptides into the polymer chain but also the choice of the peptide sequence varies strongly in the literature.^18,19^ The ECM is a highly complex, hydrated network that consists of various macromolecules, such as collagens, proteoglycans, and adhesive glycoproteins including fibronectin, laminin and vitronectin. The binding of the adhesive proteins to integrin is also important for mechano-signalling and leads to biochemical-signalling responsible for the regulation of cell attachment, spreading, proliferation and differentiation.^20–24^ The alginate polymer chains can be modified directly with these cell-adhesive peptides or indirectly over modified polyethylene glycol (PEG) moieties, which are also widely used in hydrogel systems for biofabrication studies.^25–27^ Until today various peptide sequences were used to specifically modify natural inert biomaterials, such as ALG.^28,29^ For example, the sequence tyrosine–isoleucine–glycine–serine–arginine (YIGSR, Peptide A), found in laminin,^30^ was shown to promote neuronal cell adhesion and differentiation,^31^ neuronal outgrowth^32^ and nerve regeneration.^33^ It was also studied for endothelisation^34–36^ and has been reported to induce attachment and spreading of many cell types.^37^ Furthermore, the synthetic peptide sequence arginine–arginine–glutamine–threonine–alanine–tryptophane–alanine (RRETEWA, Peptide B) derived from an adhesive glycoprotein^38^ was shown to induce osteogenic differentiation^39^ and upregulation of bone morphogenetic protein^40^ in mesenchymal stem cells. Lastly, the sequence isoleucine–lysine–valine–alanine–valine (IKVAV, Peptide C) is also found in laminin,^41^ although it is localised on a different chain than YIGSR. It was shown to promote cell attachment and neurite outgrowth^41^ and it also promotes angiogenesis^36,42,43^ and induces osteogenesis.^44^ However, in most studies, only one peptide sequence was used, which is contrary to the composition of the natural ECM, where multiple adhesive sequences are presented in a complex manner. Studies that do use multiple peptides often report a synergistic effect of peptides leading to a better cell-material interaction compared to the single sequences, respectively. For example, Peptide A and Peptide C act synergistically together with other peptides on β-cell function^45^ and promote neurite growth.^46^ However, to the best of the authors' knowledge, the combination of more than two peptides for biofabrication approaches has not been investigated.

Therefore, this study aimed to engineer cell-adhesive properties in peptide-modified alginate-based bioinks for biofabrication. The primary challenge was the coupling of Peptide A, Peptide B, and Peptide C to ALG or ADA *via* two different chemical modifications. We compared procedures and efficiency to evaluate these modifications. In this regard, the bioinks underwent a comprehensive analysis of material characteristics, including oxidation degree, molecular weight, substitution efficiency, degradation, and stiffness. Additionally, we compared the sterile syringe and drop-on-demand (DoD) printer techniques for the production of sterile hydrogel capsules. Finally, we investigated the cell-spreading behavior of NIH/3T3 fibroblasts in ALG and ADA hydrogel capsules, examining the influence of different peptide sequences coupled *via* carbodiimide or Schiff base reaction. Peptide A, Peptide B, and Peptide C, both individually and in combination, were studied to quantitatively evaluate the corresponding cell response, specifically the cell aspect ratio.

## 2. Materials and methods

Sodium alginate was obtained from JRS Pharma (PH163S2, J. Rettenmaier & Söhne GmbH + Co KG, Germany). According to previous studies, the molecular weight is 216 kg mol^−1^ and the dynamic viscosity of a 1% w/v solution at 20 °C is *η*_0_ = 0.93 Pa s.^47^ 4-arm poly(ethylene glycol)maleimide (PEG-Mal) with a chain length of 10 kDa was obtained from SINOPEG (China). Deuterium oxide (D_2_O, 99.9%), anhydrous calcium chloride (CaCl_2_) and sodium metaperiodate (NaIO_4_) were supplied from Sigma Aldrich (Germany). Spectrum Labs dialysis tubes (molecular weight cut off: 6–8 kDa) were purchased from Fisher Scientific GmbH (Germany). EDC·HCl (99%) and sulfo-NHS sodium salt (98%) were purchased from Carbolution Chemicals GmbH (Germany). MES monohydrate (≥99%) was purchased from Merck KGaA (Germany). All peptides (C-GG-YIGSR-NH_2_, C-GG-RRETAWA-NH_2_ and C-GG-IKVAV-NH_2_ shortly described as YIGSR, RETEWA and IKVAV) with amine and carboxyl acid end groups were purchased from Chempeptide Limited (China). Hanks balanced salt solution (HBSS, with calcium and magnesium) and Dulbecco's phosphate buffered saline (DPBS, no calcium, no magnesium) were supplied from Thermo Fisher (Germany). For cell culture, DMEM was supplemented with 1% pen/strep and 1% glutamine obtained from Thermo Fisher (Germany) and 10% bovine calf serum (BCS) from Corning (Germany). Calcein-AM, DAPI, trypsin-EDTA, trypan blue, penicillin–streptomycin, l-glutamine, BCS, sodium pyruvate and rhodamine phalloidin were obtained from Thermo Fischer (Germany). Ultra-pure water was produced using a Milli-Q system (Merck, Germany). NIH/3T3 fibroblast cells were obtained from ATCC (USA). Sterile filters (0.45 μm and 0.22 μm) were purchased from Carl-Roth (Germany). Hydroxylamine hydrochloride (Ph. Eur.) in 70 g l^−1^ methanol was purchased from Th. Geyer GmbH & Co. KG (Germany).

All bioinks used for the present work are summarised in Tables 1 and S1† with detailed information about the corresponding composition.

**Table 1:** Theoretical/ideal degree of substitution for carbodiimide-modified ALG-Peptide as well as ADA-Peptide, for Schiff base-modified ADA + Peptide, for Michael addition-coupled PEG-Peptide and Schiff-base-modified ADA + PEG-Peptide bioinks using certain peptide quantities of Peptide A, Peptide B and Peptide C determined for 1 g ALG, ADA or PEG(Mal)_4_

Synthesis method	Peptide coupled to compound	Peptide quantity [mg]	Peptide quantity [μmol]	Label
Carbodiimide reaction	Peptide A coupled to ALG	10.18	12.56	ALG-Peptide A
Carbodiimide reaction	Peptide B coupled to ALG	13.91	12.56	ALG-Peptide B
Carbodiimide reaction	Peptide C coupled to ALG	9.34	12.56	ALG-Peptide C
Carbodiimide reaction	Peptide A coupled to ADA	11.51	14.19	ADA-Peptide A
Carbodiimide reaction	Peptide B coupled to ADA	15.72	14.19	ADA-Peptide B
Carbodiimide reaction	Peptide C coupled to ADA	10.55	14.19	ADA-Peptide C
Schiff base reaction	Peptide A coupled to ADA	11.51	14.19	ADA + Peptide A
Schiff base reaction	Peptide B coupled to ADA	15.72	14.19	ADA + Peptide B
Schiff base reaction	Peptide C coupled to ADA	10.55	14.19	ADA + Peptide C
Michael addition	Peptide A coupled to PEG(Mal)_4_	20.27	25.00	PEG-Peptide A
Michael addition	Peptide B coupled to PEG(Mal)_4_	27.63	25.00	PEG-Peptide B
Michael addition	Peptide C coupled to PEG(Mal)_4_	18.62	25.00	PEG-Peptide C
Schiff base reaction	PEG-Peptide A coupled to ADA	94 189	87.12	ADA + PEG-Peptide A
Schiff base reaction	PEG-Peptide B coupled to ADA	94 873	85.43	ADA + PEG-Peptide B
Schiff base reaction	PEG-Peptide C coupled to ADA	94 042	87.52	ADA + PEG-Peptide C

### 2.1 Synthesis of ADA

**Fig. 1:**
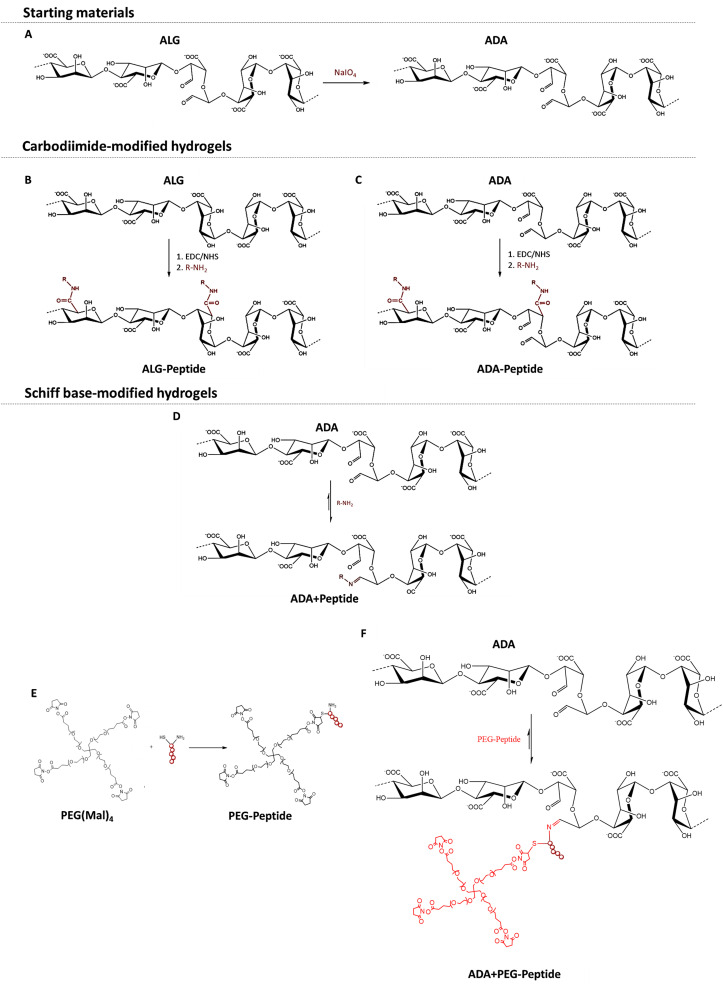
Oxidation of ALG using NaIO_4_ yielding ADA with two aldehyde groups per oxidized monomer (A), carbodiimide reaction of peptides with ALG (B) and ADA (C) using EDC·HCl as a reaction agent and Sulfo-NHS as a stabilizer, Schiff base reaction of peptides with ADA leading to a reversible bond between the peptide and ADA (D), Michael addition between amine end groups of PEG(Mal)_4_ and the sulfhydryl end groups of the peptide yielding PEG-Peptide (E), Schiff base formation between amine end groups of PEG-Peptide and the aldehyde groups of ADA yielding in ADA + PEG-Peptide with a reversible imine bond (F).

To obtain ADA with a theoretical degree of oxidation of approximately 13%, ALG was oxidised using sodium meta periodate (NaIO_4_) according to Karakaya *et al.*^11^ (Fig. 1A). Briefly, 10 g ALG was dispersed in 50 ml ethanol, to which 1.337 g NaIO_4_ dissolved in 50 ml ultra-pure water was added while stirring. The reaction was quenched after 6 h by the addition of 10 ml ethylene glycol. After further stirring for 30 min, the mixture was left to stand for 10 min, which allowed the ADA product to settle to the bottom. The liquid layer was decanted, and the sediment was dissolved in 300 ml ultra-pure water. The obtained viscous solution was filled into dialysis tubes (molecular weight cut off: 6–8 kDa) and dialyzed for 4 days against 17 l of ultra-pure water with daily water changes. The synthesis, as well as the dialysis, were done under strict dark conditions to prevent the light-induced degradation of periodate being an undesired side reaction of the oxidation. After dialysis, the product was frozen at −20 °C and then lyophilised. The ADA synthesis was performed in triplicate which were combined after dialysis to obtain comparable degrees of oxidation and molecular weights between different batches.

### 2.2 Synthesis of compounds

All bioink compositions described in this publication were synthesised from the educts described in the Materials and methods section. The detailed synthesis of Alg peptide, ADA peptide, ADA+ peptide, PEG peptide and ADA + PEG peptide is described in sections 2.3–2.6.

### 2.3 Synthesis of ALG-Peptide and ADA-Peptide

For the irreversible coupling of the peptides to ALG and ADA, the well-known carbodiimide reaction was used, as described by Rowley *et al.*^48^. In this regard, peptides were covalently coupled to ALG by reacting their amine groups with the carboxylic acid groups of ALG or ADA, which results in an irreversible amide bond formation (Fig. 1B and C). In general, a monomer activation of 5% was used, which corresponds to a ratio of 1 : 20 EDC to ALG monomers. EDC·HCl was stabilized with Sulfo-NHS in a ratio of 2 : 1. ALG-Peptide A, ALG-Peptide B, ALG-Peptide C and ALG-Peptide ABC with a theoretical degree of substitution of 0.25% was synthesized using Peptide A (*M*_W_: 810.92 g mol^−1^), Peptide B (*M*_W_: 1105.22 g mol^−1^) Peptide C (*M*_W_: 744.95 g mol^−1^) shown in Table 1. For the preparation of ALG-Peptide ABC, each peptide was coupled separately to ALG by adding a Peptide A, Peptide B and Peptide C containing solution subsequently after each other to the ALG solution aiming for a final peptide substitution degree of 0.75% (3 × 0.25% per peptide). Briefly, 1 g ALG was dissolved in 100 ml 0.1 M MES/0.3 M NaCl buffer at pH 6.5.

This buffer was produced by adding 21.33 g MES monohydrate and 17.53 g NaCl to 1 l ultra-pure water. The pH value was adjusted using a 1 M NaOH solution. To the dissolved ALG solution, 48.14 mg EDC·HCl and 24.07 mg Sulfo-NHS dissolved each in 1 ml MES buffer were added. The solution was allowed to stir for 15 min. Then, the desired amount of peptide dissolved in 1 ml buffer solution was added (see Table 1) and the solution was stirred for a further 24 h. Then, all products were filled into dialysis tubes (molecular weight cut off: 6 kDa–8 kDa) and dialyzed for 3 days against 6 l of ultra-pure water with daily water changes. After dialysis, all compounds were frozen at −20 °C and then lyophilized, respectively. The same procedure was applied for ADA which has a lower molecular weight compared to ALG, because the sodium ions being present in ALG were removed during the purification step of ADA. Therefore, different quantities of the reactants were needed: For 1 g ADA, 54.42 mg EDC·HCl and 30.82 mg Sulfo-NHS were used for the synthesis of ADA-Peptide A, ADA-Peptide B, ADA-Peptide C and ADA-Peptide ABC with a theoretical degree of substitution of 0.25% using the same method and the Peptide A, Peptide B and Peptide C (see Table 1).

For ALG-Peptide ABC and ADA-Peptide ABC all three peptides with a respective degree of substitution of 0.25% for each peptide and a final peptide concentration of 0.75% per gram ADA were used.

### 2.4 Synthesis of ADA + Peptide

For the second method, all peptides were coupled to ADA *via* Schiff base formation,^11^ where the N-terminal amine group from the peptide forms a Schiff base with an aldehyde group from the ADA (Fig. 1D). For the reversible Schiff base reaction, 10 mg ADA was dissolved in 2 ml DPBS buffer, and then the desired amount of peptide dissolved in 100 μl DPBS buffer was added. The mixture was stirred at 37 °C for 10 minutes which is sufficient to facilitate the Schiff base formation.^11^ For all cell experiments, freshly produced hydrogel solutions were used, but for further, larger scale experiments, all ADA solutions containing peptides can be lyophilised after the stirring process and stored just like the carbodiimide coupled products. To compare the carbodiimide reaction to the Schiff base reaction, ADA was coupled with Peptide A, Peptide B and Peptide C using concentrations yielding the same theoretical degrees of substitution per gram ADA (Table 1). For ADA + Peptide ABC all three peptides with a respective degree of substitution of 0.25% for each peptide and a final peptide concentration of 0.75% per gram ADA were used.

### 2.5 Synthesis of PEG-Peptide

For the synthesis of peptide-modified PEG-Peptide compounds, the Michael addition reaction according to Guo *et al.*^49^ was used, whereby peptides were irreversibly bonded to PEG(Mal)_4_ moieties (Fig. 1E). Briefly, 1 g of PEG(Mal)_4_ was dissolved in 3 ml DPBS, followed by the addition of the desired amount of peptide dissolved in 4 ml DBPS under vigorous stirring and the exclusion of light. After 3 h, the reaction was stopped, the solution was transferred into dialysis tubes (molecular weight cut off: 6–8 kDa) and dialyzed for 24 h against 5 l of ultra-pure water with daily water changes. After dialysis, all compounds were frozen at −20 °C and then lyophilised, respectively. Table 1 depicts the concentrations of Peptide A, Peptide B and Peptide C yielding in a theoretical single-arm substitution of PEG(Mal)_4_ for PEG-Peptide A, PEG-Peptide B and PEG-Peptide C. In a last approach, all three peptides were bonded to one corresponding PEG(Mal)_4_ compound by the addition of Peptide A, Peptide B and Peptide C in the same concentration occupying theoretically 3 of 4 arms of PEG(Mal)_4_.

### 2.6 Synthesis of ADA + PEG-Peptide

PEG-Peptide was conjugated to ADA by Schiff base formation according to Karakaya *et al.*^11^ as described previously yielding ADA + PEG-Peptide (Fig. 1F). In brief, ADA was dissolved in DPBS and subsequently mixed with PEG-Peptide also dissolved in DPBS under continuous stirring for 10 min at 37 °C which is known to be sufficient for the formation of Schiff bases. Table 1 depicts the concentrations of used PEG-Peptide for 1 g ADA.

### 2.7 Chemical characterization

#### 2.7.1 Degree of oxidation

To confirm the success of the oxidation of ALG, the degree of oxidation of the final ADA was determined using the method of Zhao *et al.*^50^ with slight changes according to Karakaya *et al.*^11^ Even though, this study was focused on oxidised dextran, the described method was also used before to determine the degree of oxidation in other periodate-oxidised polymers, such as cellulose,^51^ xanthan gum,^52^ pullulan^53^ and ALG.^54,55^ Therefore, this protocol was slightly modified and adapted for the determination of the degree of oxidation of ADA, which is defined as the percentage of oxidised monomeric units in the polymer. Briefly, 100 mg lyophilised ADA was dissolved in 7.5 ml ultra-pure water. To this, 7.5 ml of a 0.25 M hydroxylamine hydrochloride (HA·HCl) solution at pH 5 was added leading to a partial precipitation of ADA. Then, the sample was put into a shaking incubator at 60 °C and removed after 15 min to vigorously stir them for further 5 min leading to a complete redissolving of ADA. Subsequently, the solution was allowed to react for 6 h under the same conditions in a shaking incubator at 60 °C under continuous shaking. After cooling down the ADA solution to room temperature, it was titrated back to pH 5 using a 0.1 M NaOH solution. The volume consumption of NaOH was noted. Three replicates (*n* = 3) were used for each sample and the average NaOH consumption was determined. Unoxidised ALG served as a negative reference which was also analysed in triplicate. The degree of oxidation was calculated according to the following equation:1
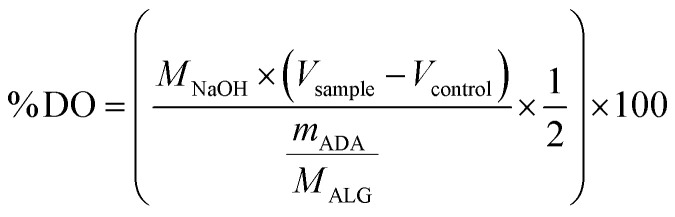
with *M*_NaOH_ as the concentration of NaOH, *V*_sample_ as the consumed volume of NaOH for ADA, *V*_control_ as the volume of NaOH for ALG, the *M*_W_ of an alginic acid monomer as *M*_Alg_ and *m*_ADA_ as the initial weighted sample.

By multiplying the volume of NaOH with the concentration of NaOH, the molar concentration of consumed NaOH is obtained. This is divided by the molar concentration of the sample, which is obtained by dividing the mass of the sample with the molecular weight of the monomeric alginate subunit (176.139 g mol^−1^). The resulting percentage is the percentage of aldehyde groups. To obtain the final degree of oxidation, this value needs to be divided by two, because one oxidised monomeric unit contains two aldehyde groups.

#### 2.7.2 Ellman assay

To determine the success of the Michael addition between thiol groups of Peptide A, Peptide B, Peptide C and the malein-amide groups of PEG(Mal)_4_, the Ellman assay according to Martin *et al.*^56^ with slight changes was performed. Briefly, 250 μl of the starting peptide solution was extracted before starting the synthesis and mixed with 50 μl Ellman reagent (DTNB) and 2500 μl DPBS to produce a reference solution. To confirm the success of the Michael addition, 250 μl of the peptide coupled PEG(Mal)_4_ solution was extracted before dialysis and mixed with 50 μl DNTB solution and 2500 μl DPBS. Then, 10 μl of the reference as well as the sample solution were diluted in 1000 μl DPBS and analysed using a UV-vis spectrometer (Specord 40, Analytik Jena GmbH, Germany) in the absorption range of 300–700 nm to compare the absorbances before and after the peptide addition.

#### 2.7.3 Fourier transform infrared spectroscopy

To confirm differences in the chemical structure of all modified products, FTIR absorbance spectra were taken using a Shimadzu IRAffinity-1S FTIR spectrophotometer (Shimadzu Corp, Japan). All samples were analysed as dry products after lyophilisation. Native ALG, unmodified ADA or PEG(Mal)_4_ were used as a corresponding reference. Further, 40 spectra per sample were recorded in the range of 400 to 4000 cm^−1^ with a resolution of 4 cm^−1^ using the Happ–Genzel apodization method.

#### 2.7.4 Nuclear magnetic resonance

NMR measurements were conducted to determine the modifications of ALG and ADA using a Bruker Avance spectrometer (Bruker Biospin GmbH, Germany). For this purpose, 10 mg of each product was dissolved in 1 ml D_2_O overnight, respectively. Then, liquid state ^1^H-NMR spectra were recorded with a triple resonance measuring probe (PATBO500S1BB-H/F-D05ZFB) operating at the Larmor frequencies of 500 MHz on ^1^H nuclei. All samples were measured at 50 °C with 64 scans, an acquisition time of 3.1457 s, 2 s pulse delay and a pulse angle of 30°.

#### 2.7.5 Gel permeation chromatography

To analyse the *M*_W_ of all polymers, gel permeation chromatography (GPC) was performed. The GPC system consisted of a pump and injection system (Viscotek GPCmax, Malvern Instruments Ltd, UK) combined with a refractive index (RI) detector (Viscotek VE3580, Malvern Instruments Ltd, UK), multiple angle light scattering detector (Viscotek SEC-MALS 20, laser wavelength 660 nm, Malvern Instruments Ltd, UK) and a viscosity detector (Viscotek 270, Malvern Instruments Ltd, UK). For the measurements, an aqueous eluent 0.1 M NaNO_3_ with 0.02% NaN3 for conservation at a flow rate of 0.7 ml min^−1^ with 100 μl injection volume was used. For separation, two polymethyl methacrylate columns (A6000, Malvern Instruments Ltd, UK) and a precolumn all kept at 35 °C were applied. Each sample was injected 5 times (*n* = 5). The decrease of *M*_W_ was calculated as follows:2
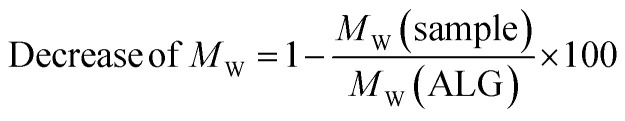
with *M*_W_ (ALG) as the molecular weight of ALG and *M*_W_ (sample) as the molecular weight of the sample.

#### 2.7.6 Hydrogel and bioink preparation

Hydrogel solutions with concentrations of 0.5% (w/v) of ALG-Peptide, ADA-Peptide, ADA + Peptide and ADA + PEG-Peptide were produced by adding 20 mg of the corresponding polymer to 4 ml DPBS followed by stirring overnight and sterile filtration using a 0.45 μm filter for sterile conditions. After processing, all hydrogels were cross-linked with 0.25 M CaCl_2_ for 30 min. For *in vitro* studies, NIH/3T3 fibroblast cells (1 million per ml) were mixed with the bioink solution under sterile conditions and subsequently transferred into a sterile cartridge or syringe.

#### 2.7.7 Degradation studies

For the investigation of the degradation rate of all materials, their behaviour in DMEM according to Karakaya *et al.*^11^ was analysed. In brief, round-shaped cylindric films were produced as described previously and stored in DMEM at 37 °C, 95% humidity and 5% CO_2_ in an incubator, respectively. Then, these films were weighted after certain time points, whereas the values were noted. The degradation rate was calculated according to the following:3
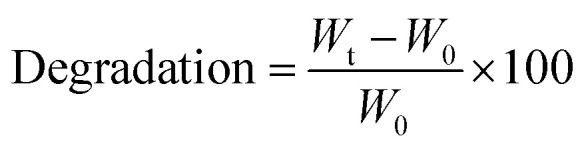
with *W*_t_ as the weight of the sample and *W*_0_ as the initial weight.

#### 2.7.8 Mechanical analysis using compression tests

The effective stiffness (Eff. Stiffness) was analysed using a microtester device (CellScale, Canada) to investigate the stiffness of all hydrogels after crosslinking with CaCl_2_ according to Hahn *et al.*^57^ For this purpose, cylindrical hydrogel films with a diameter of approximately 8 mm and a height of 2 mm were produced using a silicone mould. Briefly, a silicone mould with round gaps (diameter: 8 mm, height: 4 mm) was placed in a Petri dish filled with 50 μl of the corresponding hydrogel solution. Then, the Petri dish was closed and placed at −20 °C for 10 min. Afterwards, the frozen hydrogels in the gaps were cross-linked with 0.25 M CaCl_2_ for 30 min, removed and washed twice with ultra-pure water. Three round-shaped cylindrical samples (*n* = 3) were punched out of the hydrogel film using a biopsy puncher with a diameter of 4 mm. Samples were placed each in a water bath under the compressor beam of the microtester and compression tests were conducted using 5 cycles of 4 min as loading and unloading time, 10 s as pausing time and a force of 100 N. Furthermore, the cross tool was loaded with a loading rate of 0.25 mm min^−1^ without preloading. As a starting distance, 1.5 mm at the first point of material contact was set and the values for Eff. Stiffness were determined from the slope of the force/deformation curve in the region of 2–10% strain.

#### 2.7.9 Rheological investigations

Rheological measurements were performed by using the Discovery HR-3 rheometer (TA Instruments, New Castle, USA). Briefly, 0.7 ml of the bioink was placed on the bottom plate of the rheometer and by lowering the top steel plate with a diameter of 40 mm, a thin film of bioink with a thickness of 0.3 mm was generated. The measurements of the rheological properties as a function of shear rate (2–500 s^−1^) were conducted at 20 °C using the mentioned parallel plates. For the crosslinked samples thin films made of the corresponding hydrogel were prepared and measured with the same setting as stated before followed by the addition of CaCl_2_ (0.25 M). After 30 min of crosslinking, all rheological investigations were performed. A solvent trap was used to prevent evaporation during the measurements. The rheological analysis was performed in triplicates (*n* = 3).

### 2.8 Biofabrication

#### 2.8.1 Manually fabricated capsules

First, to generate samples as sterile as possible, only tools received sterile from the supplier (Sarstedt, Germany) were used. According to the authors, by manually ejecting bioink droplets from a sterile syringe the potential for contamination was as low as possible. For this purpose, firstly, the bioink was prepared by mixing the NIH/3T3 cell pellet (1 million per ml) into the hydrogel using a high viscous pipette as described previously (step 1, Fig. S1†). After transferring the bioink into a sterile syringe (volume: 3 ml), it was manually ejected into a 0.25 M CaCl_2_ crosslinker bath resulting in instantly forming hydrogel capsules. The crosslinker solution was not stirred and only the tip of the syringe was manually moved over the bath (step 2, Fig. S1†). After 10 min of crosslinking, all capsules were filtered using a cell strainer and washed twice with sterile ultra-pure water (step 3, Fig. S1†). In the final step, all capsules from the same composition were gathered in one cell strainer which was placed in a 6-well plate, poured over with DMEM (4 ml per well) and incubated under sterile conditions at 37 °C 95% humidity and 5% CO_2_ in an incubator, respectively. Moreover, light microscopy images of the capsules were taken using a bright field microscope (Primo Vert, Carl Zeiss, Germany) to analyse the shape details of the fabricated capsules.

#### 2.8.2 Drop-on-demand printing

For the uniform production of droplets, the BioX 3D printer (Cellink, Sweden) equipped with a DoD printing head was used. In brief, sterile bioinks were transferred into cartridges, and by applying a cycle time of 350 ms, an open time of 1 ms, a printing speed of 5 mm s^−1^ and a pressure of 15 kPa, hydrogel droplets were ejected through a needle with a diameter of 300 μm. The droplets were either directly ejected into a beaker filled with 0.25 M CaCl_2_ or printed directly on a glass Petri dish followed by the crosslinking with CaCl_2_ for 30 min. Afterwards, all hydrogels were filtered and washed twice with sterile ultra-pure water. Lastly, droplet diameters and geometries were analysed by bright field microscopy (Primo Vert, Carl Zeiss, Germany).

#### 2.8.3 *In vitro* studies

For all cell experiments, the cell line NIH/3T3 (murine embryonal fibroblasts) was used which was continuously cultured in T75 cell culture flasks using high-glucose DMEM supplemented with 10% (v/v) BCS, 4 mM l-glutamine, 1 mM sodium pyruvate and 100 U ml^−1^ penicillin–streptomycin. The cells were incubated in a controlled atmosphere of 95% relative humidity, 5% CO_2_ and 37 °C and were subcultured twice a week at about 75% confluence. For bioprinting experiments, cells were counted with a Neubauer counting chamber. For this, 50 μl cell suspension was mixed with 50 μl of a 0.2% trypan blue solution and then added to the counting chamber. The cell suspension was split into portions of 1 million cells per 1 ml hydrogel in 15 ml falcon tubes. They were centrifuged at 350 rpm for 5 min, after which the supernatant was removed. The cell pellet was later mixed with the hydrogel using a high viscous pipette which was used for the fabrication of cell-loaded capsules.

After 7 days of incubation, cells in the corresponding bioinks were stained with Calcein-AM, fixed with 3.75% formaldehyde, stained further with rhodamine phalloidin, DAPI and epifluorescence images were taken using a fluorescence microscope (FM) (Zeiss, Germany). Ten images were taken per composition. All cells were investigated later using the software ImageJ (version 1.52n) where the aspect ratio of cells (*n* = 50) was estimated by the ratio of its size in *x* and *y* dimension and was calculated by the quotient of *x* and *y*.

### 2.9 Statistical analysis

One-way analysis of variances was used for the determination of the mean differences using the software Origin 2020 (ANOVA, OriginLab Corporation, USA). ANOVA and pairwise comparison of the means was performed using Bonferroni correction to test significances. For each experiment, several replicates (*n*) were investigated. All data were shown as means ± SD and intervals of confidence as *p < 0.05, **p < 0.01 and ***p < 0.001 for the determination of statistical significance levels.

## 3. Results and discussion

### 3.1 Chemical characterisation

**Fig. 2:**
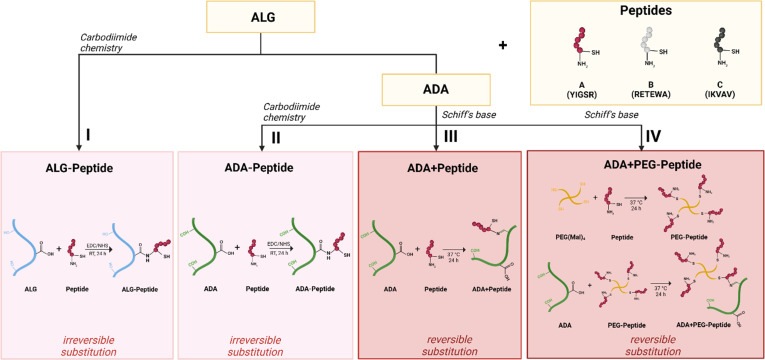
Schematic illustration of bioinks design: Irreversible substitution of a peptide to ALG using carbodiimide chemistry (I), irreversible substitution of peptide to ADA using carbodiimide chemistry (II), reversible substitution of peptide to ADA using Schiff base chemistry (III), reversible substitution of PEG-peptide to ADA using Schiff base chemistry (IV).

After ALG and ADA were modified with Peptide A, Peptide B, Peptide C, or Peptide ABC *via* carbodiimide (Fig. 2I and II) or Schiff base chemistry (Fig. 2III and IV), the chemical composition of all compounds was investigated confirming the success of each synthesis.

#### 3.1.1 Degree of oxidation

To confirm the successful transformation of ALG to ADA, the degree of oxidation was determined according to the modified protocol of Zhao *et al.*^50^ The principle of this assay lies in the interaction of HA·HCl with aldehyde groups of ADA forming oximes, which liberate HCl decreasing the pH value of the solution. Following this theory, per mole aldehyde group one mole HCl is liberated which is determined using the eqn (1). The results proved the oxidation of ALG to ADA by revealing a degree of oxidation of 11% (±0.33). This is reported by previous studies^11^ and proves the synthesis was carried out in sufficient conditions preventing the decomposition of NaIO_4_ and the successful interaction of it with ALG resulting in ADA.

#### 3.1.2 Ellman assay

**Fig. 3:**
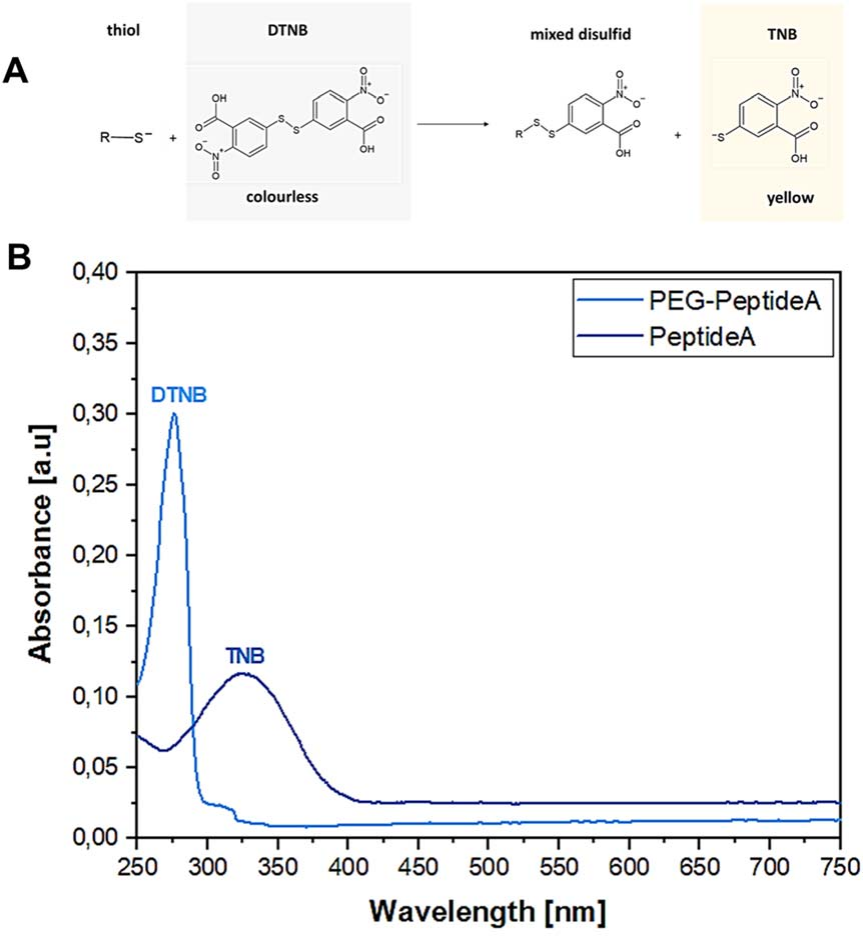
Reaction between thiol-containing compounds and DNTB results in a yellow TNB product used as a detection indicator (A), absorption spectra showing the shift of DTNB from 325 nm to 275 nm representing TNB, confirming the success of the Michael addition between peptide A and PEG(Mal)_4_ resulting in PEG-peptide A (B).

The Ellman assay was carried out according to Winther *et al.*^58^ to show the success of the covalent coupling between the thiol end groups of peptides and the malein-amide groups of PEG(Mal)_4_ moieties.^59^Fig. 3 depicts the UV-vis absorbance spectra of Peptide A before and after the addition of PEG(Mal)_4_ confirming the successful Michael addition between the peptide and PEG(Mal)_4_ yielding PEG-Peptide A. DNTB in combination with Peptide A led to the formation of mixed disulfide and 2-nitro-5-thiobenzoic acid (TNB) (Fig. 3A) indicated by a colour change of the solution from transparent to yellow resulting in absorbance at 325 nm (Fig. 3B). After PEG(Mal)_4_ was added to the Peptide A containing solution and reacted for 3 h, a second sample was taken out and investigated before dialysis yielding an absorbance at 275 nm. This proved that all present peptides were bound to PEG(Mal)_4_ and no free peptides were available which could potentially react with DTNB leading to TNB.

### 3.2 FTIR studies

**Fig. 4:**
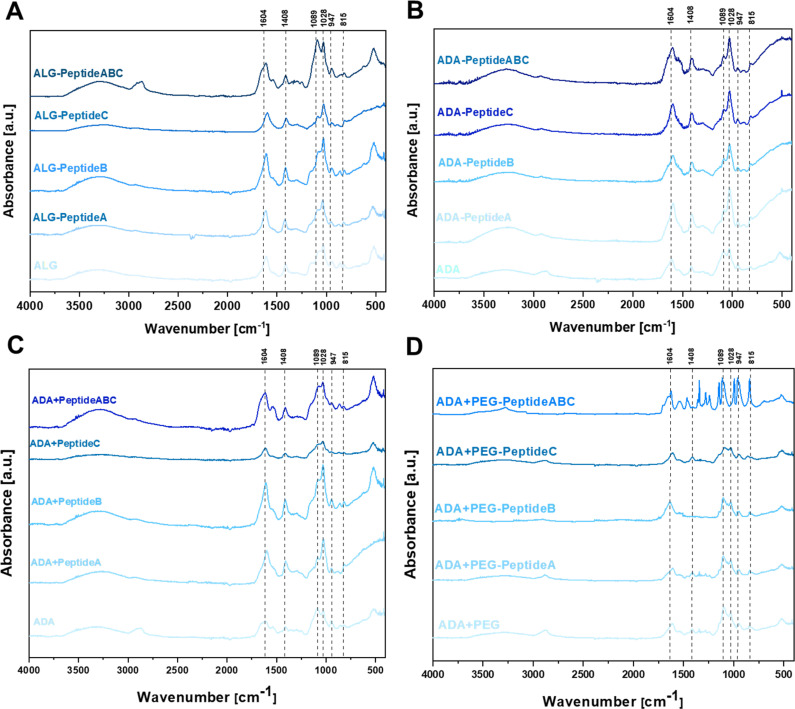
FTIR spectra of ALG and modifications as ALG-Peptide A, ALG-Peptide B, ALG-Peptide C and ALG-Peptide ABC using carbodiimide chemistry (A), ADA and the peptide-modified ADA-Peptide A, ADA-Peptide B, ADA-Peptide C and ADA-Peptide ABC using carbodiimide chemistry (B), ADA and the peptide-modified ADA + Peptide A, ADA + Peptide B, ADA + Peptide C and ADA + Peptide ABC using Schiff base formation (C) and ADA and the peptide-modified ADA + PEG-Peptide A, ADA + PEG-Peptide B, ADA + PEG-Peptide C and ADA + PEG-Peptide ABC using Schiff base formation (D).

To confirm the changes in the molecular structure of the initial and final hydrogels, FTIR studies were conducted. Fig. 4 depicts the FTIR spectra of carbodiimide-modified ALG (A), carbodiimide-modified ADA-Peptide (B), Schiff base-modified ADA + Peptide (C) and Schiff base-modified ADA + PEG-Peptide (D), all coupled with either Peptide A, Peptide B, Peptide C or Peptide ABC. The spectra of ALG (Fig. 4A) and ADA (Fig. 4B) depict peaks at 1598 cm^−1^ and 1407 cm^−1^ representing the COO^−^ stretching, respectively. Further, C–O stretching was found at 1301 cm^−1^ and 945 cm^−1^ and 1024 cm^−1^. C–O–C stretching was observed, whereas C–C stretching was found at 1078 cm^−1^. All these peaks are characteristic of ALG or ADA and have been studied many times in previous studies.^8,16,60–62^ No C=O stretching of the aldehyde group in the region of 1720–1740 cm^−1^ could be observed which confirms the absence of these groups caused by the formation of intramolecular hemiacetals.^61,63–65^ Depending on the coupled peptide, changes in the FTIR spectra of native ALG and modified ALG-Peptide as well as ADA and modified ADA-Peptide compounds were expected, but as there are no significant differences observed, no conclusions about the carbodiimide synthesis can be drawn from the FTIR data. However, it should be mentioned that small amounts of peptides were used for the synthesis of ALG-Peptide and ADA-Peptide and since the actual degree of peptide substitution is not known, only traces of peptides might have been coupled to ALG or ADA, which explains why no characteristic peaks for the corresponding peptides were obtained. Fig. 4C depicts the FTIR spectra of ADA, ADA + Peptide A, ADA + Peptide B and ADA + Peptide C, all modified *via* the Schiff-base reaction. The comparison of pure ADA and the peptide-modified compounds revealed no significant changes in the spectra.

Only the spectra of ADA + Peptide ABC depict a shift of the ADA peak which was supposed to be at 1598 cm^−1^ broadened and shifted to left indicating the Schiff base formation.^66,67^ Also, Liu *et al.*^68^ used a Schiff base coupled ADA-Peptide at a concentration of 40 mg peptide per gram ADA and reported the formation of a visible peak which they assigned to the C=N stretching of the Schiff base. However, this peak shift could not be observed for the single-coupled ADA + Peptide A, ADA + Peptide B and ADA + Peptide C compounds. Even though no peak indicating the presence of any amine could be obtained, it is known that all Schiff base-modified ADA + Peptides certainly contain peptides since no further purification step was performed after the synthesis. The reason for the absence of peptide peaks might be the low concentration present in the final hydrogels which is below the reported concentration of Liu *et al.*^68^ The data shown in Fig. 4D do not reveal any further differences between ADA + PEG and the Schiff base-modified ADA + PEG-Peptide A, ADA + PEG-Peptide B and ADA + PEG-Peptide C which might be attributed to the low peptide concentration in the corresponding final hydrogel. However, considering the spectrum of the multiple-coupled ADA + PEG-Peptide ABC a significant change in the FTIR spectra attributed to the presence of peptides could be seen which confirms a successful Michael addition. To conclude, the FTIR spectra were not sufficient to confirm the single peptide modification in all cases. Also, no significant changes in the spectra could be observed for the carbodiimide-modified ALG-Peptide and ADA-Peptide compounds. Only for higher concentrations, present in the Schiff base-modified ADA + Peptide ABC and ADA + PEG-Peptide ABC compounds containing higher peptide concentrations, significant differences were observed.

### 3.3 NMR studies

**Fig. 5:**
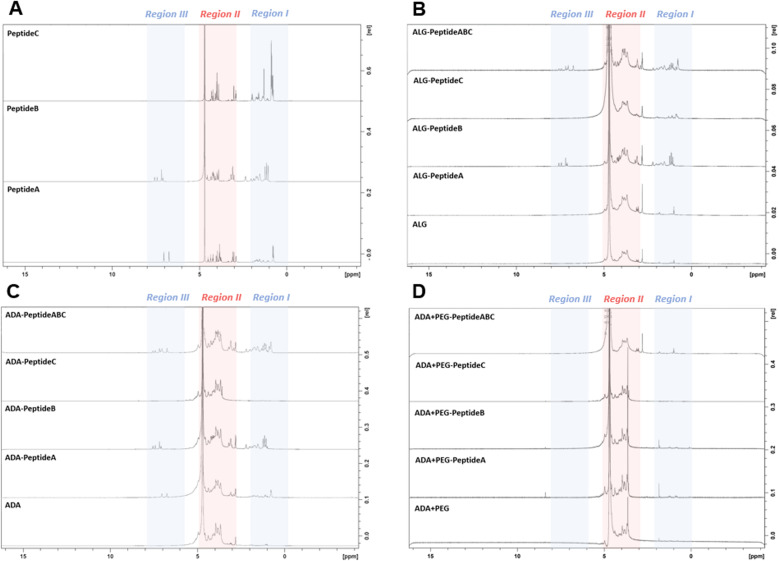
NMR spectra of Peptide A, Peptide B and Peptide C (A), ALG and modifications as ALG-Peptide A, ALG-Peptide B, ALG-Peptide C and ALG-Peptide ABC using carbodiimide chemistry (B), ADA and the peptide-modified ADA-Peptide A, ADA-Peptide B, ADA-Peptide C and ADA-Peptide ABC using carbodiimide chemistry (C), and ADA and the peptide-modified ADA + PEG-Peptide A, ADA + PEG-Peptide B, ADA + PEG-Peptide C and ADA + PEG-Peptide ABC using Schiff base formation (D).

To confirm the presence of peptides in the final bioinks, NMR studies of all peptide-modified ALG, ADA and ADA + PEG hydrogels were conducted. NMR analysis of the Schiff's base modified ADA + Peptide A, ADA + Peptide B, ADA + Peptide C and ADA + Peptide ABC were neglected since no further purification step after the synthesis was performed and therefore the presence of the corresponding peptides in the final bioinks was certainly ensured. Fig. 5A depicts the NMR spectra of Peptide A, Peptide B and Peptide C which were divided into three regions. While Peptide A and B show signals in region I (0–2 ppm), region II (3–5 ppm) and region III (6–8 ppm), Peptide C shows significant signals only in regions I and II. Since the signals in region II of all peptides completely overlap with the signals of ALG and the strong solvent signal, this range is neglected for all following compositions. Fig. 5B demonstrates the NMR spectra of ALG and the corresponding carbodiimide-modified equivalents all coupled with either Peptide A, Peptide B, Peptide C or Peptide ABC. Even though, ALG-Peptide A and ALG-Peptide C indicate weak signals in region I, no signals could be observed for ALG-Peptide A in region III. Therefore, the presence of the corresponding peptides could not certainly be proven for these bioinks. In contrast to that, explicit signals in regions I as well III could be obtained for ALG-Peptide B and ALG-Peptide ABC confirming certainly the presence of peptides in the final bioinks. However, the exact number of coupled peptides could not be identified using NMR spectroscopy. Further, Fig. 5C shows the NMR spectra of ADA and the corresponding carbodiimide-modified equivalents all coupled with either Peptide A, Peptide B, Peptide C or Peptide ABC. The results indicate weak signals in regions I and III for ADA-Peptide A and explicit signals for ADA-Peptide C and ADA-Peptide ABC proving the success of the carbodiimide reaction. However, for ADA-Peptide C no signals could be observed as to why the coupling of this peptide seemed to be rather unsuccessful. Lastly, all peptide-coupled ADA + PEG compositions were investigated. The results in Fig. 5D revealed no signals for any of the bioinks in regions I and III. The reason for that could be the extremely low final peptide concentrations in the final hydrogels which were not detectable anymore.

### 3.4 GPC studies

**Fig. 6:**
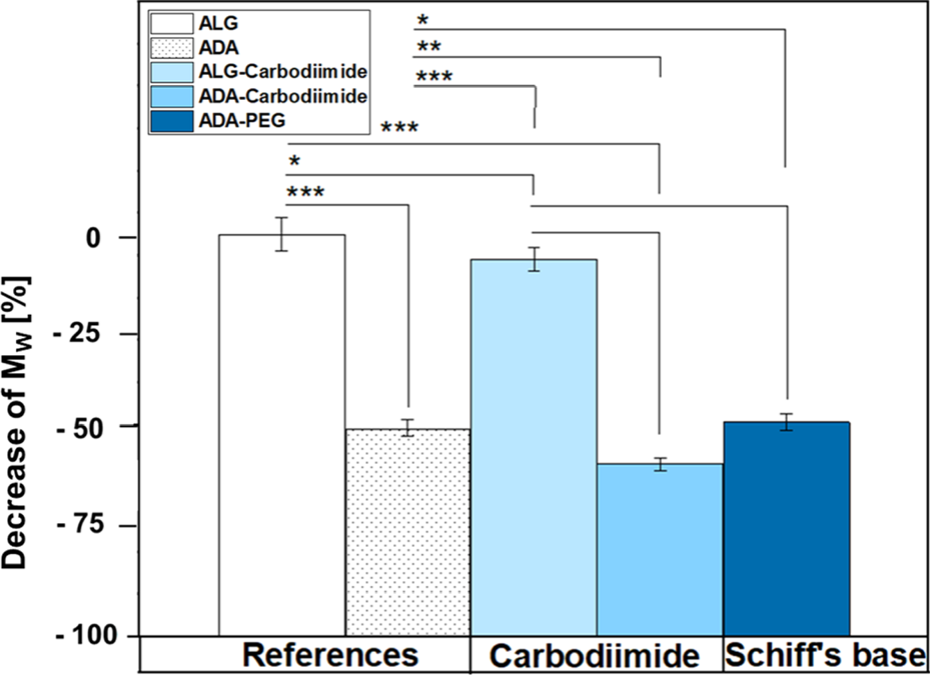
Decrease of *M*_W_ depending on ALG using the GPC results of the references ALG and ADA as well as the final compounds ALG-Carbodiimide, ADA-Carbodiimide and ADA + PEG which underwent the conditions of the carbodiimide and Schiff's base reaction, respectively.

Fig. 6 depicts the decrease of *M*_W_ values of ADA and the corresponding synthesis products depending on the starting material ALG. A drastic decrease was observed for ADA (−51.6%) compared to ALG which can be explained by the radical depolymerization during the oxidation with NaIO_4_ yielding in the decrease of the chain length depending on the amount of used oxidation reagent.^9^ Furthermore, slight weight loss was observed for ALG-Carbodiimide (−7.4%) and ADA-Carbodiimide (−60.0%). The reason for that may be the reaction conditions during the carbodiimide reaction. It is known that alginate chains are unstable in aqueous media and can undergo cleavages induced by hydrolysis in an acidic environment.^69^ The synthesis medium maintained a slightly acidic pH of approximately 6.5, which contributed to minimal molecular weight (*M*_W_) reductions.

Specifically, ALG-Carbodiimide exhibited a modest decrease of −7.4%, while no significant changes were observed for ALG (0%) and ADA-Carbodiimide (−60.0%) compared to their respective control groups, ALG and ADA (−51.6%). Additionally, ADA + PEG demonstrated a relatively minor *M*_W_ reduction of −49.5% compared to ADA (−51.6%). This minimal impact on *M*_W_ can be attributed to the mild conditions of the Schiff's base reaction and the short reaction time.

### 3.5 Eff. Stiffness analysis

**Fig. 7:**
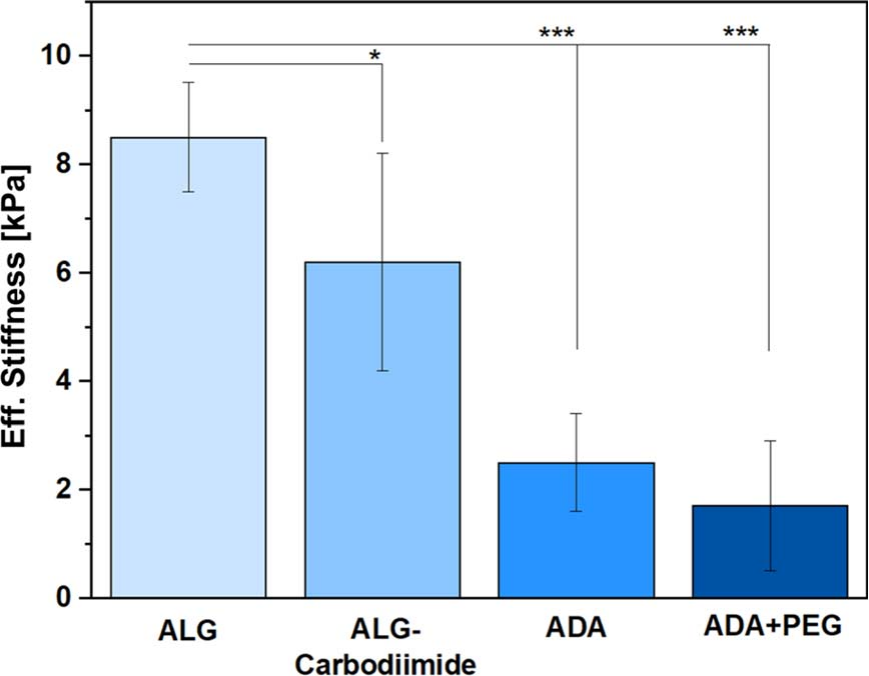
Eff. Stiffness of ALG, ALG-Carbodiimide, ADA and ADA + PEG after crosslinking with 0.25 M CaCl_2_ for 30 min.

Since the amounts of peptides used for this study were so minor, only the ALG, ALG-Carbodiimide, ADA and ADA + PEG (after crosslinking with CaCl_2_) were investigated in terms of Eff. Stiffness using a micro tester device. The results (Fig. 7) revealed that ALG showed the highest values with approximately 8.5 kPa (±1.9) followed by ALG-Carbodiimide with 6.2 kPa (±3.8), ADA with approximately 2.3 kPa (±1.7) and ADA + PEG with approximately 2 kPa (±2.1). This is following the expectation since ALG contains the highest number of guluronic acids (G-acids) which are needed for the crosslinking with CaCl_2_ resulting in a dense polymer network due to a stiff egg-package of crosslinked ALG.^16^ The Eff. Stiffness of ALG-Carbodiimide is lower than the one of ALG since the acidic conditions during the synthesis led to chain breakages. Further, during the oxidation of ALG, G-acids are preferentially attacked by the oxidation reagent. Thus, the number of free G-acids in ADA is lower compared to pristine ALG. In addition, the polymer chains of ALG were decreased due to the unwanted side reaction induced by radicals during the synthesis.^9^ Taking these two factors into account, the decrease of the Eff. Stiffness from 8.5 kPa to 2.3 kPa was expected. Moreover, no significant difference was observed for the stiffness of ADA and ADA + PEG. The amount of PEG(Mal)_4_ is comparably low in contrast to the ratio of ADA and is additionally not involved in the cross-linking of ADA with CaCl_2_. Therefore, the presence of PEG(Mal)_4_ is not beneficial for crosslinking, and it probably is incorporated between the crosslinked ADA chains, leading to cavities in the polymer network.

These cavities are known to decrease the Eff. Stiffness of crosslinked hydrogels, which could be observed through the slight decrease from approximately 2.3 kPa for ADA to approximately 2.0 kPa for ADA + PEG on average.^11^

### 3.6 Degradation studies

**Fig. 8:**
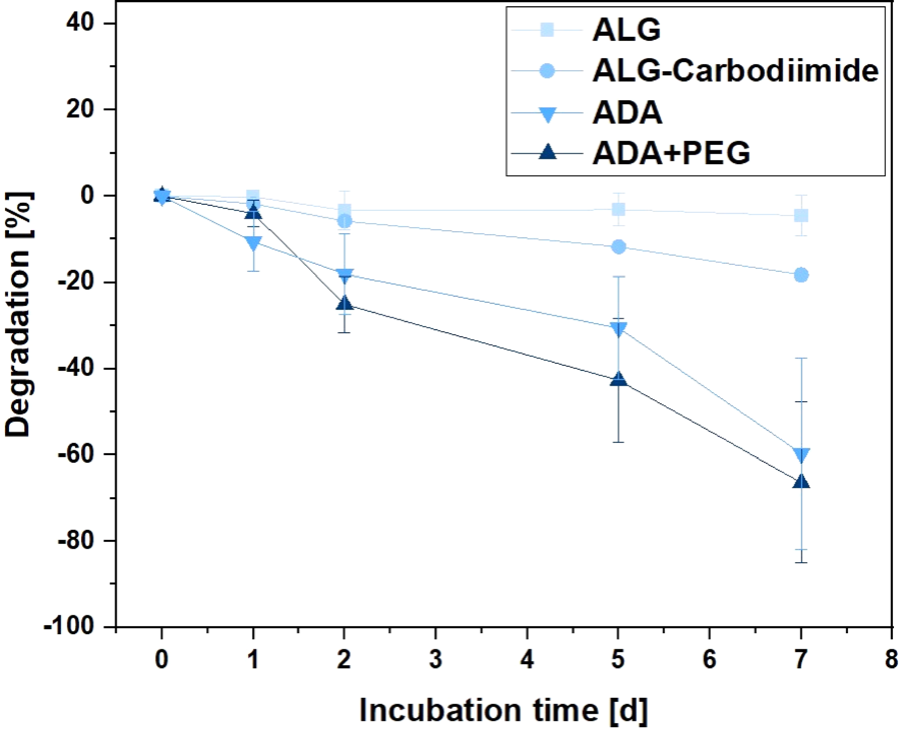
Degradation of ALG, ALG-Carbodiimide, ADA and ADA + PEG incubated in DMEM at 37 °C for 7 days.

Hydrogels are recognised for their degradation in aqueous media, breaking down into distinct components that create cavities within the 3D polymer network. These cavities provide a conducive environment for the growth of incorporated cells.^70^ This property is crucial for various applications of bioinks, including drug delivery and cell-encapsulated implantations.^71,72^ Therefore, the degradation rates of ALG, ALG-Carbodiimide, ADA and ADA + PEG were investigated over the incubation time of 7 days to observe differences. The effect of peptides is so small that it could be neglected and was therefore not further analysed. Fig. 8 depicts the degradation rate of the hydrogels showing the slowest degradation rate of −3% for ALG after 7 days of incubation. This behaviour is by the literature claiming that ALG starts to significantly degrade after weeks at ambient conditions.^73^ Only for the modification of ALG to ALG-Carbodiimide and ADA, increased degradation of approximately −18% and −58% was observed. The lower interaction between CaCl_2_ and the limited G-acids as well as the shorter polymer chains of ALG-Carbodiimide and ADA led to a weaker polymer network compared to the ones of ALG. Furthermore, the monomers of ADA containing aldehyde groups are more likely to be attacked by water and degrade *via* hydrolysis.^8^ This is the reason why ADA showed higher degradation rates compared to ALG. Nevertheless, the highest degradation rate was observed for ADA + PEG with approximately 63% after 7 days of incubation. The primary component in this blend system is ADA, contributing to comparable degradation behaviors in both hydrogels. In contrast to the homogeneous network present in pure ADA, the ADA + PEG blend incorporates uncrosslinked PEG(Mal)_4_ moieties, weakening the 3D structure. This alteration influences the decomposition behavior in the medium, resulting in the highest degradation rate observed in ADA + PEG.

### 3.7 Rheological investigations

**Fig. 9:**
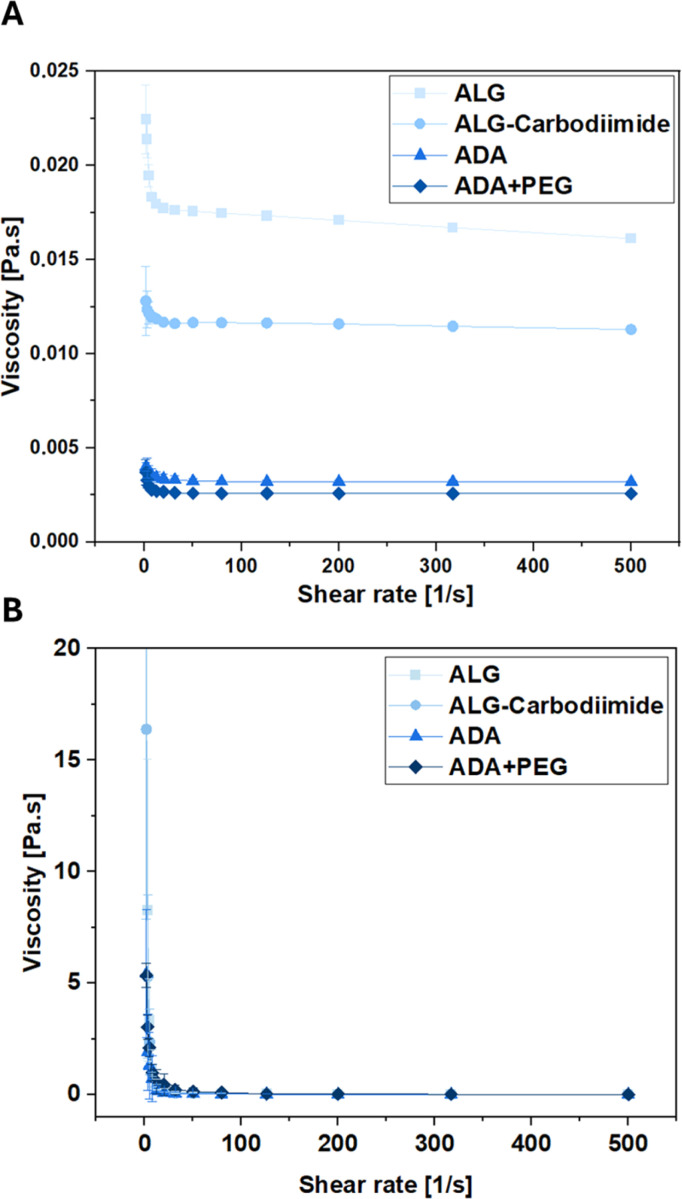
Viscosity as a function of shear rate of ALG, ALG-Carbodiimide, ADA and ADA + PEG determined by rheological analysis before (A) and after crosslinking (B).

To investigate the conditions experienced by cells encapsulated in the bioinks during the printing process, the viscosities of ALG, ADA and ADA + PEG hydrogels were investigated before cross-linking. The corresponding results depicted in Fig. 9 reveal that ALG showed the highest viscosity, which can be deducted from the long polymer chains and therefore the high *M*_W_ of ALG. The viscosity values of ALG-Carbodiimide and ADA are significantly lower due to the decreased polymer chains induced by the acidic treatment during the carbodiimide reaction and the depolymerization during the oxidation reaction.^74^ Lastly, the ADA + PEG hydrogel system revealed the lowest viscosity values. The reason for that may be the PEG(Mal)_4_ moieties, which seemed to hinder the polymer alignment decreasing the viscosity slightly compared to pure ADA. Further, rheological analysis of the hydrogels performed after the crosslinking with CaCl_2_ revealed no significant difference as shown in Fig. 9B and S2.† This showed that the oxidation of ALG as well as the further modification of ADA with PEG(Mal)_4_ moieties did not affect the crosslinking capability of the remaining G-acids within the ADA chain, exhibiting the same viscosity as native ALG.

### 3.8 Biofabrication

**Fig. 10:**
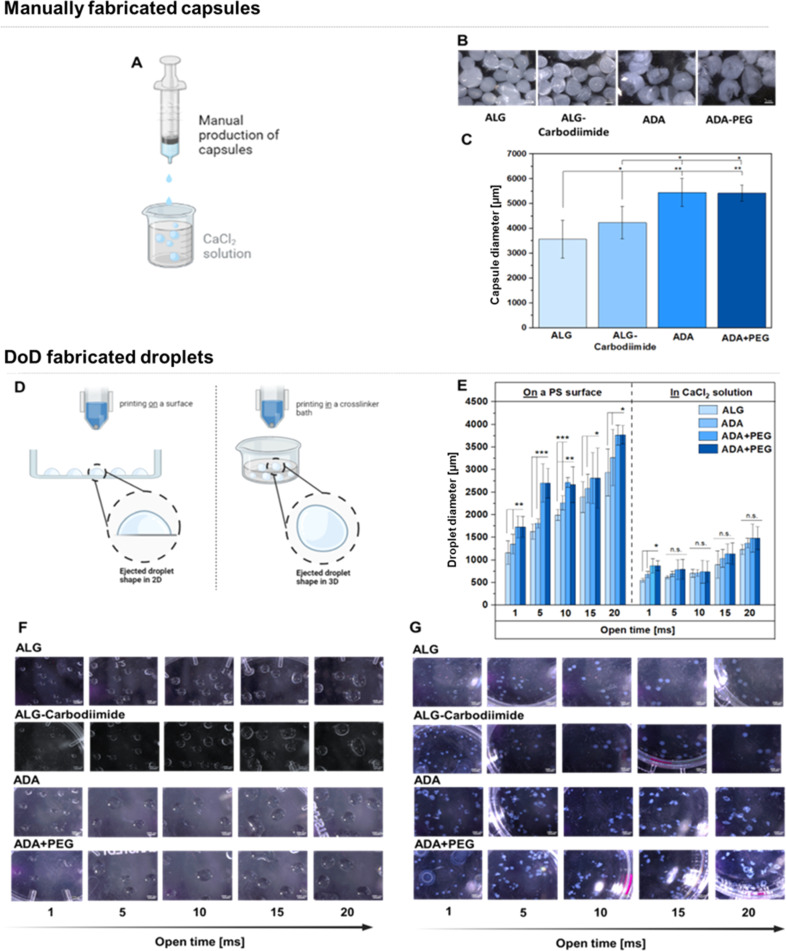
Set up for the manual production of capsules using a hydrogel filled in a syringe and ejected in a crosslinker solution consisting of CaCl_2_ (A), LM images of ALG, ALG-Carbodiimide, ADA and ADA + PEG capsules (B), determined diameters of manually fabricated capsules made of ALG, ALG-Carbodiimide, ADA and ADA + PEG (C), set up for the fabrication of droplets using a DoD printer ejecting hydrogel droplets in a crosslinker solution resulting in round shaped droplets (left) or on the surface of a glass Petri dish followed by crosslinking with CaCl_2_ resulting in a half ovate droplet shape (D), determined diameters of 3D printed ALG, ALG-Carbodiimide, ADA and ADA + PEG droplets using different open times (1–20 ms) (E) LM images of printed ALG, ALG-Carbodiimide, ADA and ADA + PEG droplets printed either on PS (F) or in a CaCl_2_ solution (G).

**Fig. 11:**
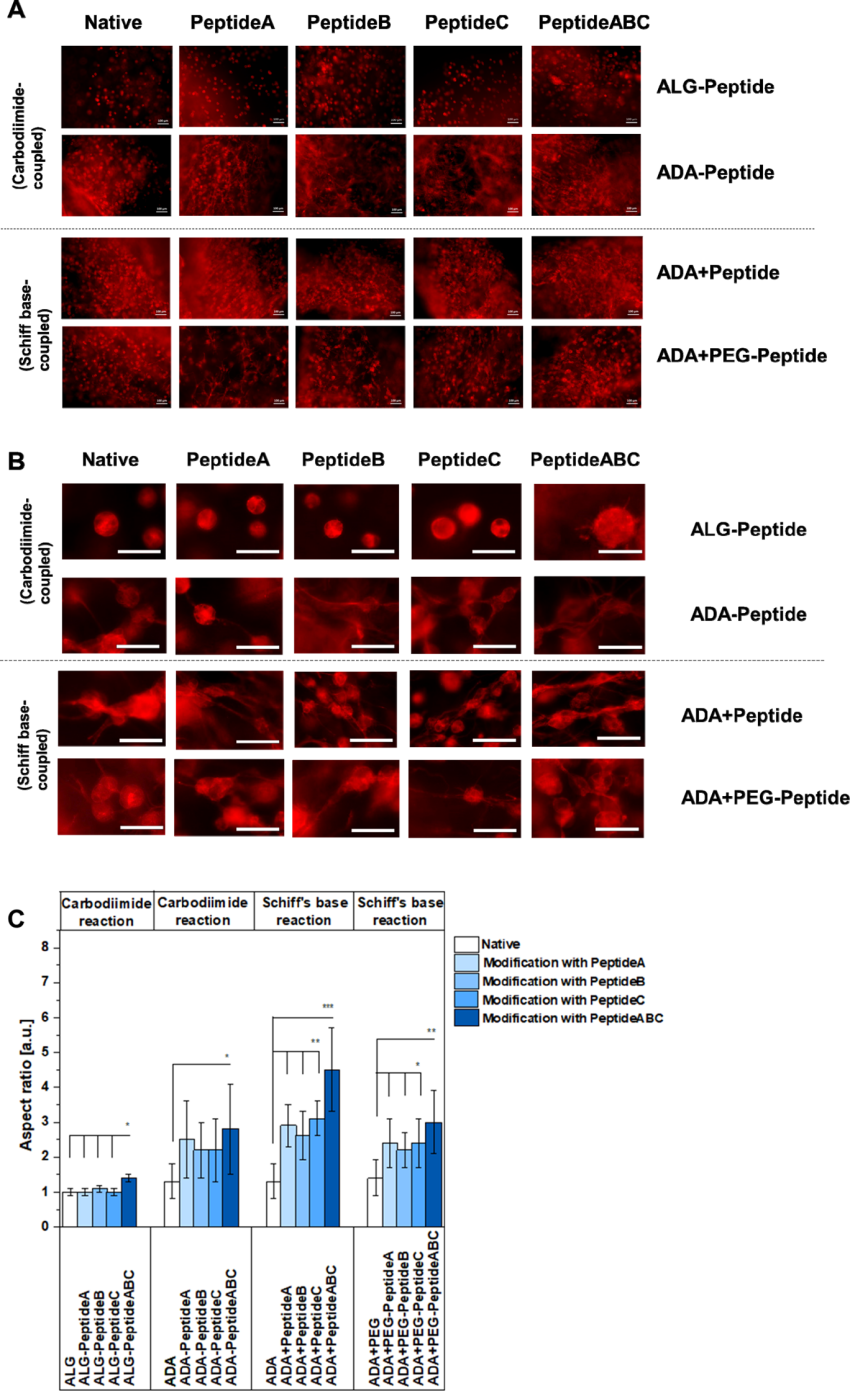
FM images of NIH/3T3 cells grown in ALG-Peptide, ADA-Peptide, ADA + Peptide and ADA + PEG-Peptide bioinks after 7 days of incubation and stained with rhodamine-phalloidin for F-actin (cytoskeleton), scale bar: 100 μm (A) and scale bar: 50 μm (B), aspect ratio of NIH/3T3 cells in ALG-Peptide, ADA-Peptide, ADA + Peptide and ADA + PEG-Peptide bioinks after 7 days of incubation, *n* = 50 (C). * = *p* < 0.05, ** = *p* < 0.01, *** = *p* < 0.005.

To fabricate hydrogel capsules or droplets, two distinct approaches were employed and compared. Firstly, manual production was executed using exclusively sterile tools to mitigate any risk of contamination. This method involved the use of a sterile syringe and a CaCl_2_ crosslinker solution (Fig. 10A). Light Microscopy (LM) images unveiled the macrostructure of the hydrogel capsules, portraying round shapes with smooth surfaces for ALG and ALG-Carbodiimide (Fig. 10B). In contrast, ADA capsules appeared more fragile, exhibiting round but uneven shapes. This fragility can be attributed to the weak crosslinking potential of ADA with CaCl_2_ compared to ALG, resulting in more delicate structures.^16^ A similar observation was noted for ADA + PEG capsules, primarily composed of ADA. The determined diameters (Fig. 10C) showcased the smallest values (approximately 3500 μm) for ALG capsules, followed by ALG-Carbodiimide capsules (approximately 4200 μm). This aligns with expectations and can be attributed to the robust interaction between CaCl_2_ and G-acids in ALG, leading to a stiffer network and smaller capsule diameters. ALG-Carbodiimide capsules were slightly larger due to the smaller ALG chains caused by acidic conditions during carbodiimide synthesis, resulting in weaker interactions with CaCl_2_. It's worth noting that the manual ejection of droplets led to a higher standard deviation. Conversely, ADA and ADA + PEG capsules exhibited significantly higher diameters (approximately 5300 μm) due to the weak crosslinking of ADA with CaCl_2_, indicating a less dense polymer network with larger diameters. No significant difference between ADA and ADA + PEG was observed, as ADA dominated the properties of both compositions in the final hydrogel system. In a second approach, droplets were produced using a 3D DoD printer, where hydrogel droplets were mechanically ejected either into a CaCl_2_ solution (Fig. 10D, left) or directly onto a glass Petri dish, followed by crosslinking with CaCl_2_ (Fig. 10D, right), employing different cycle times (1–20 ms). The smallest droplets (approximate diameter: 1200 μm) were observed for ALG at a cycle time of 1 ms, followed by ALG-Carbodiimide (approximate diameter: 1400 μm), ADA (approximate diameter: 1700 μm), and ADA + PEG (approximate diameter: 1700 μm). The highest diameter (approximately diameter: 3500 μm) was obtained with ADA + PEG and ADA at a cycle time of 20 ms (Fig. 11E, right), whereas ALG (approximate diameter: 2600 μm) and ALG-carbodiimide (approximate diameter: 3100 μm) showed smaller diameters. LM images of the hydrogel droplets also revealed an increase in diameters with the cycle time ranging from 1 ms to 20 ms (Fig. 11E). In all cases, the smallest droplets were obtained using ALG and ALG-Carbodiimide, whereas ADA and ADA + PEG showed higher diameter values for all cycle times. No significant difference between ADA and ADA + PEG was observed. Additionally, droplets printed directly into CaCl_2_ crosslinker solution (Fig. 10E, left) exhibited a similar behaviour. While ALG and ALG-Carbodiimide capsules showed the smallest diameter (approximately 500–1500 μm), ADA and ADA + PEG droplets revealed slightly higher diameters (approximately 1000–1800 μm) depending on the cycle time (1–20 ms). Comparing both setups (Fig. 10E right and left), it can be summarised that droplets ejected into a CaCl_2_ solution showed larger diameters than the ones ejected directly onto a printing surface, which can be correlated to the impact on the either soft water surface or hard printing surface. In this regard, it should also be mentioned that droplets ejected directly into a crosslinker solution were immediately crosslinked while the hydrogel droplets printed on a surface were crosslinked after the printing process was finished. Therefore, the diameter of the droplets can also be attributed to the mentioned crosslinking order. Furthermore, it is notable that the standard deviation for all compositions printed with the DoD printer (<1000 μm, Fig. 10E) was lower compared to the manually fabricated hydrogel capsules (>1000 μm, Fig. 10C). However, it should be mentioned that the DoD needle may be clogged/blocked very easily by the hydrogel and therefore several trials were needed until hydrogel droplets could be printed successfully. This can be problematic for bioprinting approaches since the sterility of the needle is not given in case of needle changes.

Therefore, the inconsistent shape resulting in various droplet diameters caused by small blockages in the needle and the maintenance of sterility conditions during the DoD printing process are the main drawbacks of this method. In conclusion, the manual printing method was not sufficiently useful for cell experiments requiring long-time sterility.

### 3.9 Cell-bioink interaction

The cell spreading was analysed using the FM images of cells in the corresponding bioinks stained with rhodamine-phalloidin. Fig. 11A and B show the representative FM images of NIH/3T3 cells incubated for 7 days in native ALG, ADA, ADA + PEG as a negative reference and their modified versions using Peptide A, Peptide B, Peptide C and Peptide ABC coupled by either carbodiimide or Schiff base reaction, respectively, to compare the efficiency of the synthesis route and the corresponding peptides. Cells in peptide-modified ALG bioinks seemed not to spread very significantly showing representative round cells in Fig. 11B and S3,† an aspect ratio values of 1 for Peptide A-, Peptide B- and Peptide C-modified ALG hydrogels depicted in Fig. 11C. Compared to that, cells in ALG-Peptide ABC seem to slightly spread in ALG-Peptide ABC as representatively shown in Fig. 11B. Further, Fig. 11C reveals an aspect ratio of 1.3 for ALG-Peptide ABC. In addition, the strong changes in cell morphology with the development of long adhesion structures under the influence of YIGSR-modified bioinks are presented in the images in Fig. S3,† right. The comparison of these results with the ones of the NMR studies revealed a discrepancy. While the presence of peptides in ALG-Peptide B and ALG-Peptide ABC could be proven using NMR, fibroblasts in ALG-Peptide B seemed not to spread significantly higher in *in vitro* studies compared to ALG-Peptide A and ALG-Peptide C. The reason for that could be the long ALG polymer chains known not to degrade after 7 days of incubation and therefore not enable any space for the cells to grow.^70^ Moreover, cells in ADA (aspect ratio: 1.3) and ADA + PEG (aspect ratio: 1.4) spread significantly higher than in pure ALG as shown in Fig. 11A and B. This cell behaviour could be explained by the lower *M*_W_ and the consequently lower stiffness of the hydrogels being following previous data leading to a faster degradation and therefore to more space for the cells compared to native ALG.^75^ Nevertheless, the differences in aspect ratio were not significant. Although many studies have been conducted with peptide-modified ALG and ADA synthesised by carbodiimide reaction, this is the first work that directly compares these two mechanisms.^76–78^ The data of Fig. 11C clearly showed different cell behaviour between ALG-Peptide and ADA-Peptide synthesised by the carbodiimide reaction. While none of the added peptides seemed to influence the cell aspect ratio of ALG-Peptide A, ALG-Peptide B, ALG-Peptide C (aspect ratio: 1.0 in all cases) compared to native ALG (aspect ratio: 1.0), ADA-Peptide A (aspect ratio: 2.0), ADA-Peptide B (aspect ratio: 2.0), ADA-Peptide C (aspect ratio: 2.2) and ADA-Peptide ABC (aspect ratio: 2.9) showed significantly higher aspect ratio values in contrast to pure ADA (aspect ratio: 1.3). Nevertheless, various studies confirmed a successful peptide modification of ALG using this technique which was not observed by our results.^28,48,79–87^ However, the used amount of peptide for the coupling varies enormously in the literature. Also, most of the time no further information about the alginate source or any material property is given. The protocol used in this work from Rowley *et al.*^48^ reports a 78% reaction efficiency for their RGD-modified ALG, but they also used a comparable lower peptide concentration. Later work from the same authors showed a reaction efficiency of 55% and 60%.^48^ Formo *et al.*^28^ modified ALG with RGD, IKVAV and YIGSR peptides using the same general protocol as used in this work, but with 10% monomer activation instead of 5%, whereas a peptide substitution of 0.4 to 1.0% could be obtained with an initial peptide input of 5%. On the other hand, Sandvig *et al.*^15^ observed only a 2–4% reaction efficiency for their peptide-modified ALG using the same protocol with 5% monomer activation and peptide excess. In summary, it can be concluded that the initial concentration obtained from literature varies drastically as many authors reported even much lower peptide concentrations, some up to 100-fold lower than the 5% ALG-Peptide.^48,88,89^ Even if the reaction efficiency for the ALG-Peptide was worse than for the ADA-Peptide, it would be reasonable to assume that at least it would still have some effect if the modification worked. A reasonable explanation for the apparent failed synthesis could be the source of ALG since the current ALG has a comparable high *M*_W_ which could prevent any attack by other compounds. It is known that some researchers pre-treat their ALG first with gamma radiation, degrade the chains and later modify them.^67,90,91^ This would also explain why the synthesis route worked for ADA-peptides which are known to degrade during the oxidation reaction in an undesired side reaction. So, the carbodiimide reaction may have worked better for ADA, because ADA has shorter chains and thus has more rotational freedom compared to ALG,^16^ which leads to better accessibility of the carboxy groups for both reaction reactants and peptides. Therefore, it can be assumed that the efficiency of the peptide coupling to the polymer chains was more successful in the case of ADA-Peptide. Further, the results of Fig. 11C for ADA-Peptide bioinks revealed that ADA-Peptide A (aspect ratio: 2.2) showed a slightly higher aspect ratio compared to ADA-Peptide B (aspect ratio: 2.0) and ADA-Peptide C (approximately 2.0), whereas ADA-Peptide ABC (aspect ratio: 2.9) depict the highest cell aspect ratio in average. However, the differences in the aspect ratio values between the peptide-modified ADAs are not significant, so it can be said that all peptides work about equally well in inducing fibroblast spreading. Fig. 11B showed highly spread cells for all these compositions. Also, it is important to mention that this is the first time proving that Peptide B can promote fibroblast spreading as effectively as Peptide A and Peptide C. Peptide A and Peptide C are commonly used in neural cell cultures and less information about their influence on fibroblasts exists.^31,48^ To the best of the authors' knowledge, this is also the first report of coupling these two peptides to ADA using the carbodiimide reaction. Another aspect worthy to mention is the relation between the degree of substitution and cell spreading in peptide-modified ALG bioinks. For the synthesis, an equal quantity of Peptide A, Peptide B and Peptide C was used (regarding mass, not molecular weight), leading to different degrees of substitutions, whereas ADA-Peptide B had the lowest one. However, the results revealed that all modified ADA-based bioinks induced cell spreading to approximately the same degree although the standard derivation was relatively high. The reason for this was the broad distribution of cell growth in different hydrogel areas induced either by an inhomogeneous peptide modification or different mechanical properties due to an inhomogeneous crosslinking.

Moreover, the ADA-Peptide ABC led to a significantly higher aspect ratio than ADA coupled with only Peptide A, Peptide B or Peptide C, respectively. The results indicated that Peptide B and Peptide C seemed to work synergistically with Peptide A yielding a higher cell aspect ratio on average. However, the initial peptide concentration for the coupling of peptides to ADA was comparable high so large-scale applications would require a high amount of costs. In contrast to that, comparable low concentrations of peptides were needed for the synthesis of ADA + Peptide A, ADA + Peptide B, ADA + Peptide C and ADA + Peptide ABC synthesised using the Schiff base reaction. Cells incubated in these bioinks for 7 days showed highly spread cells in Fig. 11B and significantly higher aspect ratios in Fig. 11C for all modified ADA bioinks (aspect ratio between 1.5 to 4.5) compared to native ADA (aspect ratio: 1.3) and the previous investigated ADA-Peptide A, ADA-Peptide B, ADA-Peptide C and ADA-Peptide ABC. In addition to that, the results indicated clearly that fibroblasts incubated for 7 days in ADA + Peptide ABC (aspect ratio: 4.5) spread to a significantly higher degree compared to ADA coupled with only Peptide A (aspect ratio: 2.6), Peptide B (aspect ratio: 2.5) and Peptide C (aspect ratio: 3.0) using the Schiff base reaction, respectively. This also confirms the synergistic effect of Peptide A, Peptide B and Peptide C on NIH/3T3 cells leading to an advanced cell–material interaction. However, the fact that the bond between peptide and ADA is a reversible imine bond^92^ which may be attacked easily by hydrolysis leading to the release of the corresponding peptide, long-term cell analysis needs to be considered for future investigations. Additionally, the degree of substitution of ADA + Peptide A, ADA + Peptide B, ADA + Peptide C and ADA + Peptide ABC could not be determined and therefore the exact peptide concentration over the incubation time was unknown. Although it cannot be certainly verified whether all peptides formed a Schiff base, peptides are still present in the final product after 7 days. However, during medium changes, peptides could potentially be released which could not be further analysed due to the low peptide concentrations. Comparing the ADA + Peptide bioinks synthesised by Schiff base reaction with ADA-Peptide synthesised by carbodiimide reaction, it becomes clear that the difference in aspect ratio is not drastic (* = *p* < 0.05), which in return allows us to draw a conclusion about the carbodiimide reaction efficiency. The aspect ratio for the carbodiimide ADA-Peptide compositions lies in the region of the Schiff base ADA + Peptide hydrogels, why it is reasonable to assume that the actual degree of substitution for the Schiff base ADA + Peptide lies at least in the same region of one of ADA-Peptide, thus showing a high efficiency considering the initial low peptide concentration needed for the synthesis. Further, it caters to the hypothesis that the carbodiimide synthesis did not work for ALG-Peptide hydrogels because of the ALG source and not due to the peptide sequences. Lastly, cells in ADA-based bioinks coupled with peptide modified PEG moieties over Schiff base reaction were investigated. FM images of stained cells in ADA + PEG-Peptide A (aspect ratio: 2.5), ADA + PEG-Peptide B (aspect ratio: 2.4), ADA + PEG-Peptide C (aspect ratio: 2.5) and ADA + PEG-Peptide ABC (aspect ratio: 2.9) compared to pure ADA + PEG (aspect ratio: 1.4) showed an obvious spreading after 7 days of incubation (Fig. 11B and C). The determined aspect ratio values revealed a significant increase from ADA to PEG-Peptide modified compounds, whereas ADA + PEG-Peptide ABC showed a slightly higher cell aspect ratio. This confirms that PEG(Mal)_4_ moieties can be used as linkers between peptides and ADA providing a stable binding until 7 days. The results also proved that several arms of PEG(Mal)_4_ can be functionalized without inhibiting the properties of each peptide. Therefore, it can be said that the main advantage of these hydrogel systems lies in the aspect of molecular engineering which allows the precise design of the bioink enabling the controlled introduction of peptides *via* irreversible Michael addition. In the second step, PEG-Peptide was bonded to ADA over Schiff base reaction. This reversible second binding enables additional self-healing properties for the bioinks^93^ and can therefore be considered as precisely engineered smart materials.

## 4. Conclusion

In conclusion, extensive investigations were conducted on all modified bioinks, encompassing both chemical and material characterisation. However, the presence of peptides could not be confirmed by FTIR and NMR spectra due to their low quantities in the final bioink. Subsequent analyses involving degradation, viscosity, and stiffness studies unveiled pivotal insights. The acidic conditions during the carbodiimide reaction caused chain breakage in ALG, reducing the stability, viscosity and stiffness. However, material characteristics of ADA + PEG bioinks, including degradation, viscosity, effective stiffness, droplet diameters and cell aspect ratios, exhibited no statistically significant deviations from those observed in ADA. The most important point to highlight in this regard is the unique flexibility of ADA + PEG bioinks, owing to their molecularly tuneable properties, distinguishing them from the remaining bioinks. Further, in contrast to ALG, both ADA and ADA + PEG bioinks exhibit properties essential for sustained cell viability and proliferation. Schiff base coupled ADA + PEG bioinks successfully induced cell spreading, almost as effectively as Carbodiimide coupled ADA-Peptides. This is the first time that peptides were coupled to ADA *via* the Schiff base, revealing high aspect ratio values for embedded fibroblast cells. A further novel aspect of this study is the synergistic effects of combining multiple peptides, resulting in higher cell spreading compared to the individually coupled bioinks. Therefore, ADA + PEG bioinks boast an additional layer of adaptability, marked by adjustable properties, particularly when incorporating peptides with a synergistic effect. The incorporation of four-armed PEG(Mal)_4_ moieties in ADA + PEG not only facilitates precise adjustments of multiple peptides simultaneously but also enables repetitive modifications of the same peptide. Thus, these precisely engineered bioink formulations, featuring novel peptide mixtures, can be regarded as highly tuneable materials with expandable properties. Future investigations will further address these properties, considering different peptide ratios, diverse cell types, and biochemical assays, aiming to further clarify these properties and explore potential applications.

## Conflicts of interest

There are no conflicts to declare.

## Supplementary Material

RA-014-D3RA08394B-s001
