# Supplementary material for: Engineering peptide-modified alginate-based bioinks with cell-adhesive properties for biofabrication
Source: RSC Adv. 2024 Apr 26;14(20):13769–86. doi: 10.1039/d3ra08394b (PMC11046382; doi:10.1039/d3ra08394b)
Supplement: RA-014-D3RA08394B-s001 [file RA-014-D3RA08394B-s001.pdf]

Electronic Supplementary Material (ESI) for RSC Advances.
This journal is © The Royal Society of Chemistry 2024

Appendix

Tab.S1: List of investigated bioinks with detailed information about their composition.

Bioink label	Composition information
PeptideA	Peptide sequence YIGSR (C-GG-YIGSR-NH2)
PeptideB	Peptide sequence RRETEWA (C-GG-RRETAWA-NH2)
PeptideC	Peptide sequence IKVAV (C-GG-IKVAV-NH2)
PeptideABC	Peptide combination: YIGSR, RRETEWA and IKVAV
ALG	Pure alginate PH163S2
ALG-Carbodiimide	Pure alginate treated with the conditions of the carbodiimide reaction
ADA-Carbodiimide	Pure ADA treated with the conditions of the carbodiimide reaction
ALG-PeptideA	Alginate substituted with PeptideA via carbodiimide reaction
ALG-PeptideB	Alginate substituted with PeptideB via carbodiimide reaction
ALG-PeptideC	Alginate substituted with PeptideC via carbodiimide reaction
ALG-PeptideABC	Alginate substituted with PeptideABC via carbodiimide reaction
ADA	Oxidized alginate
ADA-PeptideA	ADA substituted with PeptideA via carbodiimide reaction
ADA-PeptideB	ADA substituted with PeptideB via carbodiimide reaction
ADA-PeptideC	ADA substituted with PeptideC via carbodiimide reaction
ADA-PeptideABC	ADA substituted with PeptideABC via carbodiimide reaction
ADA+PeptideA	ADA substituted with PeptideA via Schiff base reaction
ADA+PeptideB	ADA substituted with PeptideB via Schiff base reaction
ADA+PeptideC	ADA substituted with PeptideC via Schiff base reaction
ADA+PeptideABC	ADA substituted with PeptideABC via Schiff base reaction
PEG-PeptideA	PEG-MAL substituted with PeptideA via Michael addition
PEG-PeptideB	PEG-MAL substituted with PeptideB via Michael addition
PEG-PeptideC	PEG-MAL substituted with PeptideC via Michael addition
PEG-PeptideABC	PEG-MAL substituted with PeptideABC via Michael addition
ADA+PEG-PeptideA	ADA substituted with PEG-PeptideA via Schiff base reaction
ADA+PEG-PeptideB	ADA substituted with PEG-PeptideB via Schiff base reaction
ADA+PEG-PeptideC	ADA substituted with PEG-PeptideC via Schiff base reaction
ADA+PEG-PeptideABC	ADA substituted with PEG-PeptideABC via Schiff base reaction

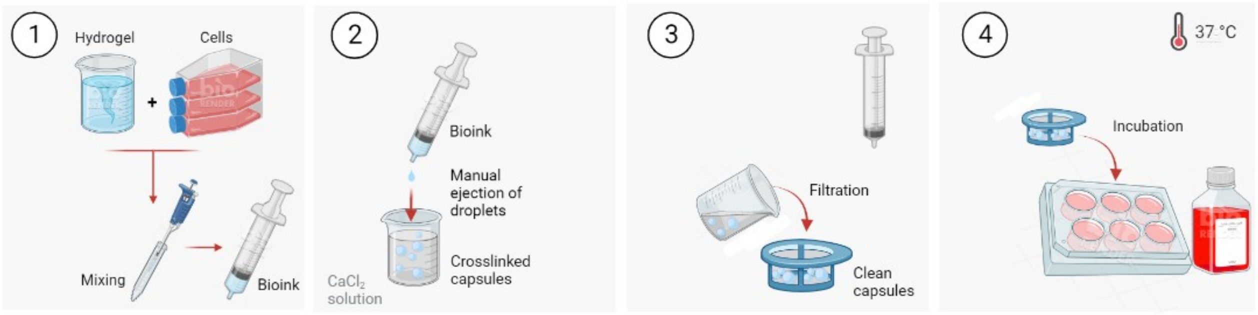

Fig. S1: Step by step procedure of manually fabricating hydrogel capsules by mixing cells into the hydrogel (step 1), ejecting the hydrogel into a $CaCl_2$ crosslinker bath using a syringe (step 2), followed by filtration using a cell strainer (step 3) and incubation in DMEM (step 4).

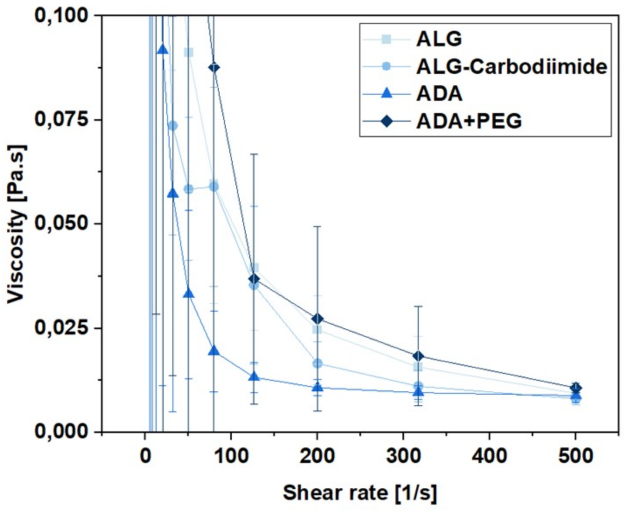

Fig. S2: Viscosity of ALG, ALG-Carbodiimide, ADA and ADA+PEG determined by rheological analysis after crosslinking (for the viscosity region 0 - 0,1 Pa.s).

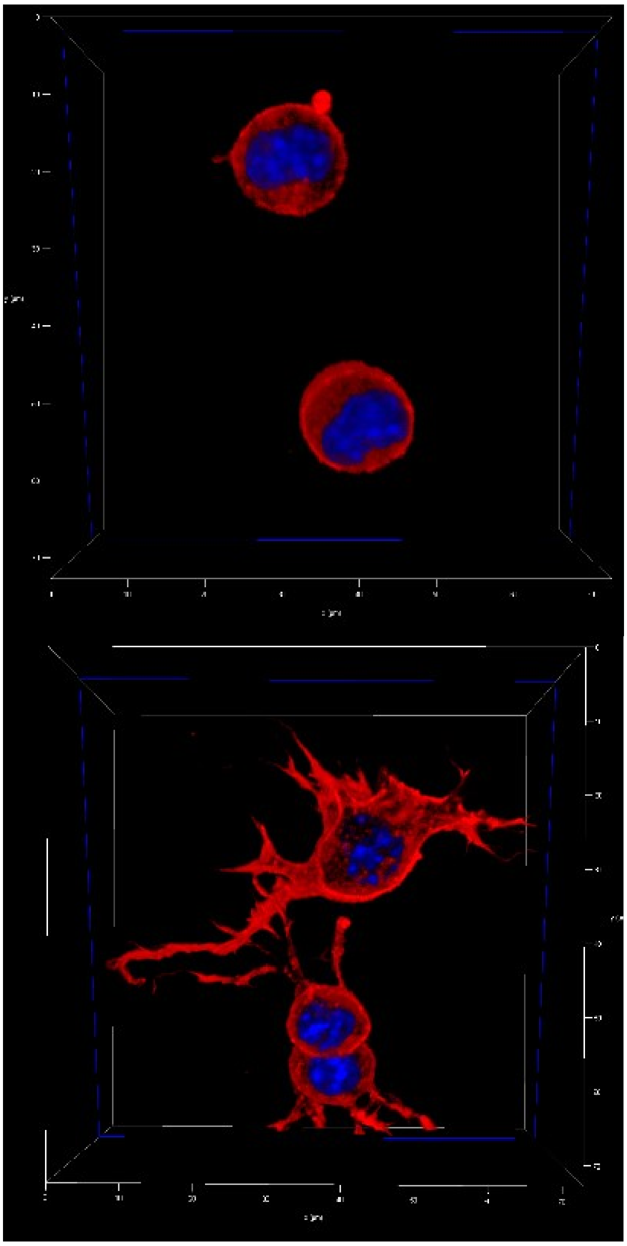

Fig. S3: Confocal images of NIH/3T3 cells grown in ALG (above) and ADA+PEG-PeptideA (below) bioinks after 7 d of incubation. Blue: DAPI, red: Phalloidin-Rhodamine. MIP. 40x/1.1 W objective at zoom

4, Z-stack with step size of 0.5 µm. Images taken using the Leica Stellaris 5 system performed by Heike Glauner, Leica Germany.
